# Injection of Chloroform in Hydrocele

**Published:** 1855-07

**Authors:** 


Injection of Chloroform in Hydrocele.—Prof. Langenbeck, of Berlin, prefers chloroform to the tincture of iodine as an injection in hydrocele. He injects from one to three drachms into the sac.

